# Network analyses of physical and psychological factors of playing-related musculoskeletal disorders in student musicians: a cross-sectional study

**DOI:** 10.1186/s12891-024-08103-8

**Published:** 2024-11-30

**Authors:** Anne-Violette Bruyneel, Florian Stern, Audrey Schmid, Nancy Rieben, Clara E. James

**Affiliations:** 1 Geneva School of Health Sciences, HES-SO University of Applied Sciences and Arts Western, Geneva, Switzerland; 2 Geneva University of Music, HES-SO University of Applied Sciences and Arts Western, Geneva, Switzerland; 3 Faculty of Psychology and Educational Sciences, University of Geneva, Geneva, Switzerland

**Keywords:** Student musicians, Playing-related musculoskeletal disorders, Physical and psychological factors, Network analysis

## Abstract

**Background:**

Young musicians starting their professional education are particularly vulnerable to playing-related musculoskeletal disorders (PRMDs). In the context of research on PRMDs, physical and psychological associated factors are frequently highlighted without investigating their complex interrelationships. The objective of this exploratory study was to examine the associations between lifestyle, music practice habits, physical and psychological variables, and PRMDs in student musicians.

**Methods:**

Students of the Geneva University of Music participated in the survey. The primary outcome was students’ PRMDs, measured with the validated Musculoskeletal Pain Intensity and Interference Questionnaire for Musicians (MPIIQM). Additionally, to investigate potential associated factors, participants completed free-form questions about lifestyle and practice habits and seven validated questionnaires: physical activity, self-rated health, psychological distress, musical performance anxiety (MPA), perfectionism, fatigue, and personality traits. After performing standard descriptive statistics, network analyses were applied to investigate the links between students' PRMDs experience and all factors.

**Results:**

Two hundred thirty-five student musicians completed the survey. 86 (37%) participants experienced PRMDs over the last 12 months. When considering all participating students, the network analysis showed the strongest association between the presence of PRMDs and the psychological distress factor. In the subgroup with students with PRMDs, the degree of pain interference with musical practice was correlated with psychological distress, MPA, self-rated health, and fatigue.

**Conclusion:**

Psychological distress is the primary factor associated with PRMDs, whereas physical factors like posture and activity show no direct link. Therefore, addressing psychological aspects is crucial for student musicians with physical disorders to provide proper prevention care.

**Supplementary Information:**

The online version contains supplementary material available at 10.1186/s12891-024-08103-8.

## Background

Musical practice at pre-professional and professional levels is a musculoskeletal activity with a high risk for health due to the intense repetition of movements, the number of practice hours and the constraining postures in a demanding cognitive and psychosocial context [1, 2]. Entrance to music universities seems a particularly critical time, as students are faced with high performance demands, increased intensity of practice and the need to work alone for long hours and finally fierce competition [3]. Hildebrandt et al. 2012 [4] found that after their first year in music university, students reported more fatigue, depression, and stage fright. Compared to students in other disciplines (e.g. science, business, social work), student musicians exhibit a far higher prevalence of musculoskeletal pain and symptoms related to stress and depression [3, 5, 6]. Nevertheless, they often evaluate their health more positively than their peers in other disciplines, as they consider pain an inherent consequence of rigorous musical training [3, 6]. Moreover, students who choose to study music as performers are often more perfectionistic and lead less active lifestyles compared to other students, focusing primarily on excellence in their field [5]. University students in fields outside stage performance who engage in regular physical activity (PA) experience fewer symptoms of depression, anxiety, and stress, improving their quality of life [7]. In contrast, student musicians face a demanding curriculum with high physical and psychological challenges. Intense training, perfectionism, peer influence and talent disparities limit opportunities for a healthy lifestyle [8, 9]. Student musicians’ situation is worsened by low economic resources, distance from family, limited access to healthcare, and lack of health knowledge [3, 9, 10]. Despite 10% of student musicians facing health issues threatening continuation of their studies [11], prevention strategies in music universities are rare [12]. Moreover, care provided by healthcare professionals is often inadequate to meet musicians' specific needs [10].

Professional and student musicians often focus on physical practice and error avoidance, while neglecting mental preparation and self-regulation. A study including 14 musicians [13] showed that varied strategies support progress better than repetitive drills, though repetition was commonly considered as most effective by the musicians (students and alumni) themselves.

Genuine interest in musicians’ health of medical practitioners and researchers emerged since the 1980s [14]. In 1998, Zaza et al. specified the notion of playing-related musculoskeletal disorders (PRMDs), defined as “pain and other symptoms which are chronic, are beyond of control for musicians, and which interfere with their ability to play their instrument at their usual level” [2]. This subjective self-appraisal allows including all pain and pain-related issues that arise during or as a result of playing an instrument, even without a diagnosis. This is crucial, as only a third of musicians with PRMDs have a medical diagnosis explaining their symptoms [15].

In studies on student musicians, 48 to 89% of participants self-reported a history of PRMDs over the last 12 months [6, 8, 16–18]. These findings are alarming insofar as the presence of PRMDs has significant repercussions on the psychological and emotional health of students [19], but also on their professional learning process [11]. Indeed, health problems are often taboo, and the precarious situation of student musicians usually leads to an initial denial of the problem [19]. However, prevalent pain can disrupt balanced movements during practice, leading to compensatory behaviors causing new PRMDs [20]. Student musicians often seek professional help too late, only when the issue severely hinders their training and performance, limiting treatment options and sometimes forcing them to reduce practice or even cease their studies [11].

PRMDs are characterized by their location, duration and intensity. The neck and shoulders are the most affected areas [21], with location varying by instrument, technique and upper limb posture [14]. Among student musicians with PRMDs, 51% reported chronic issues lasting over a year, and 31% had acute issues (less than 5 weeks) [21]. Associated factors involve a complex interaction of physical and psychological variables linked to music training and lifestyle [5]. Potential intrinsic risk factors include age, gender, previous injuries, poor physical functioning, fatigue, sleep quality, stress vulnerability, and personality traits [1, 2, 8, 22–24]. Extrinsic factors are the instrument played, age at practice onset, sedentary lifestyle, poor learning conditions and/or instrumental technique, extended practice without breaks and high physical and psychological demands [1, 5, 17]. The links between psychological factors (cognitive process, emotional regulation and coping) with physical disorders remain relatively underexplored, although this association might be decisive in understanding PRMDs and tailoring preventive actions [25]. Indeed, concerning sports, the International Olympic Committee indicated that both physiological and psychological stressors strongly impact injury prevalence [26]. In orchestra musicians, a strong relationship between music performance anxiety (MPA) and PRMDs was observed [22]. In student musicians, Ballenberger et al. [5, 8] considered the stress symptoms and depression as an important factors associated with musculoskeletal health complaints. A longitudinal study showed that mental health is satisfactory in the beginning of the first semester of the first year in music university, with a decline at the end of the second semester, without the association with PRMDs being explored [27]. Students in a music performance curriculum experience more stress and depression than those in other academic disciplines [5], but the results are similar to those of students in sport programs [28], which are also highly competitive environments. Alessandri et al. 2020 [28], showed that, compared to the general population, student musicians are more affected in terms of psychological rather than physical health. Moreover, Ioannou et al. 2015 [19] found that 40% of student musicians experienced poor mental health in presence of PRMDs. PRMDs often result from muscle tension, evidenced by increased electromyographic activity in the muscle [24], especially under MPA and fatigue [24, 29]. This muscular tension, combined with fatigue, exacerbates PRMDs, which in turn can worsen psychological health, creating a vicious cycle. In their 2023 review, Herman and Clark [30] primarily examined the debilitating aspects of MPA, the most widely studied, through Kenny’s inventory. Yet they also note that MPA can act as both an obstacle and a potential source of motivation. Habilitating MPA, also known as "performance energy", can enhance performance. The authors advocate for cognitive reframing and health-oriented psychological techniques to harness MPA’s positive aspects, encouraging musicians to embrace it as an integral part of the performance experience. Despite these insights, student musicians frequently neglect health and stress management, failing to adopt preventive strategies, especially when under constant pressure to excel [31]. In these previous studies, potential links between physical and psychological factors are rarely explored directly with PRMDs in depth, and with the pain intensity or pain interference with musical practice in student musicians.

This cross-sectional study aimed, with a network analysis approach, to 1) identify the prevalence of PRMDs among student musicians at university level, 2) assess the correlations between all physical and psychological factors with the presence or absence of PRMDs, and 3) identify in the group with PRMDs the factors associated with pain intensity and interference. Our main hypothesis was that over 30% of student musicians would present a history of PRMDs [14, 17]. Given the interplay between physical and psychological aspects of musicians' practice, a strong link was anticipated between psychological distress, perfectionism, self-rated health, MPA, and the presence of PRMDs, as well as with physical factors (fatigue, sedentary lifestyle, lack of warm-up exercises and breaks, age, gender, music training habits and sleep). This study’s originality lies in applying network analysis, a statistical approach that has never been used in previous studies, to address the limitations of traditional correlational and regression methods, which restrict analysis to pairwise relationships or a single dependent variable. Network analysis enables a more comprehensive examination of complex interconnections among all variables simultaneously, treating psychological and physical factors and life habits on the same level [32]. This method is especially valuable for identifying groupings of factors or indirect associations with PRMDs, as well as interactions between pain intensity and interference with musical practice. We conducted network analyses across all participants, on those with PRMDs, and those experiencing PRMDs that interfere with playing, revealing interaction patterns that simpler methods may overlook.

## Methods

This study is a monocentric cross-sectional design among student musicians at the “Haute Ecole de Musique de Genève” (HEM-GE), a university preparing students to become professional musicians. The full study protocol has been previously published [33]. Detailed explanations of the questionnaires and their validity are provided in this initial open access publication (pages 4–8). The full questionnaire is available in Appendix 1.

### Participants and recruitment

Recruitment was carried out among student musicians enrolled at the HEM-GE (Geneva and Neuchâtel sites). Nearly 90% of the 550 students at the HEM-GE were engaged in performing arts, with the remaining 10% pursuing studies in composition, conducting or “music and movement” departments.

#### Inclusion criteria

- Adult students (≥ 18 years old);
- Enrolled in any study year of a Bachelor's or Master's degree;
- Students able to follow the regular curriculum, and practice their instrument;
- Main discipline: instrumental or vocal department.

#### Exclusion criteria

- Enrolled in composition, conducting, or "music and movement" departments (due to less intense instrumental practice);
- Surgical interventions in the previous 12 months affecting music practice;
- Medical leave.

These criteria aimed to provide a comprehensive view of student musicians’ situations, and ensure sufficient participants for valid statistical inferences. The ethical committee (CCER Geneva 2022–02206) approved this study, in accordance with the Declaration of Helsinki. Information about the nature of the study was provided verbally by the researchers at the beginning of theoretical courses in each study year. After these information sessions, an e-mail with an information letter was sent. After a cooling-off period (> 24 h), all volunteers could sign the informed consent form and receive the link to complete the questionnaires.

### Administration of questionnaires

The RedCap platform (https://www.project-redcap.org/) was used to format and manage data collection. All questionnaires were administered in French or English versions online within one survey (Appendix 1). Each participant could pass the survey only once in self-reported mode. The estimated time to complete all questionnaires was 40 min. The online link to complete the survey was sent during a standard period within the curriculum, avoiding exams, orchestra sessions, and vacations.

### Outcomes

#### Socio-demographic information

The first set of questions consisted of three components [17]: (1) sociodemographic, health, and clinical aspects (age, sex, height, weight, manual laterality, work outside of study, health and educational history); (2) lifestyle (rest, diet, sleep, addictive behaviors); (3) musical practice habits (starting age, primary and secondary instrument, number of hours spent practicing in courses at the HEM-GE per day/week, number of hours spent practicing alone per day/week, academic level, breaks during practice, warm-up and cool-down exercises).

#### Primary outcome

The main outcome was PRMDs. The valid and reliable Musculoskeletal Pain Intensity and Interference Questionnaire (MPIIQM) was used, which was developed to measure musculoskeletal pain and pain interference in professional orchestra musicians [34]. It consists of 22 items, including a mix of formats such as “yes/no” items or numeric rating scales (0 to 10) items. The first eight items relate to musicians’ characteristics and their musical practice habits. The following 14 items focus on PRMDs (since musical practice onset, over the last 12 months, the last 4 weeks, and the last 7 days), their specific anatomic locations, their intensity, and the extent to which pain interfered with playing during practice. A higher score indicates a higher level of pain intensity or interference. Certain items were adapted from the original MPIIQM questionnaire, initially specific to professional orchestra musicians, while staying close to the original text.

#### Secondary outcome

The self-rated health (SRH) questionnaire was used to measure general health [35]. Participants needed to evaluate their present health in comparison to the past, to peers of the same age group, and assess how their health conditions affected their daily activities [17]. Each items using a three-point Likert-type scale, ranging for example from “bad” (= 1) to “good” (= 3) [33]. The scores for each of the four items were summed to give a total score ranging from 4 to 12 points (4 items × 3 points). A higher score indicates a higher level of SRH. The International Physical Activity Questionnaire-Short Form (IPAQ-SF) was used to measure participants’ PA [36]. An additional item was added to assess adherence to the WHO's recommended 150 min of weekly PA [9]. The IPAQ-SF consists of seven items using a continuous measurement scale (in days and/or hours) to assess PA, levels of sedentariness, and the intensity of PA (vigorous, moderate, or low) performed during the last seven days. The responses to these items are used to calculate the total PA in Metabolic Equivalent of Task—MET-minutes/week and to categorize individuals into different levels of PA.

The valid Kessler Psychological Distress Scale (Str/K10) assessed participants’ nonspecific psychological distress during the past four weeks [37]. It consists of 10 items about behavioral, emotional, cognitive, and psychophysiological manifestations of psychological distress. Each item was scored using a five-point Likert-type scale, ranging from "None of the time" (= 1) to "All of the time” (= 5), and the scores for each of the 10 items were summed to give a total score ranging from 10 to 50 (10 items × 5 points). Higher scores indicate higher levels of psychological distress. A 10 to 19 score represents “good mental health”, a 20 to 24 score represents “mild mental disorders”, a 25 to 29 score represents “moderate mental disorders” and a 30 to 50 score represents “severe mental disorders”.

The Kenny Music Performance Anxiety Inventory (K-MPAI-R) is a psychological assessment questionnaire designed to measure MPA among musicians [38]. The French version of the K-MPAI-R possesses good validity and reliability [39]. It includes 40 items using a seven-point Likert scale for responses, ranging from "Strongly Disagree" (= 0) to "Strongly Agree” (= 6), and the 40 item scores are summed to yield a total score ranging from 0 to 240 (40 items × 6 points). Higher scores indicate higher levels of anxiety and distress associated with musical performance. Each participant was classified according to six categories: no MPA, low MPA, average MPA, above average MPA, high MPA, extremely high MPA.

The "Perfectionism Motivation Questionnaire" (PMQ) was used to measure psychological characteristics related to the level of perfectionism and its underlying causes [33, 40]. It is a validated questionnaire with a bi-dimensional structure consisting of 25 items. The first dimension (self-determined perfectionism) consists of seven items, related to intrinsic motivation (4 items) and identified regulation (3 items). The second dimension consists of 18 items, related to introjected regulation (3 items), social external regulation (6 items), positive and negative material external regulation (3 items each) and motivation (3 items). Each item was scored using a seven-point Likert-type scale, ranging from "Does not correspond with me at all" (= 1) to "Exactly corresponds to me” (= 7), and the scores for each of the 25 items are summed up to a total score ranging from 25 to 175 (25 items × 7 points). A higher score indicates a higher level of perfectionism.

The valid Chalder Fatigue Scale was used to measure the severity of fatigue in participants [41]. This questionnaire consists of 11 items in total: four items are about physical fatigue, and seven about psychological fatigue. The response involves a four-point Likert scale, ranging from "Less than usual" (= 0) to "Much more than usual” (= 3), and the scores of all 11 items are summed to a total score ranging from 0 to 33 (11 items × 3 points). Higher scores indicate higher levels of tiredness.

The validated short form of the Big Five Inventory (BFI-10) was used to measure the five major dimensions of personality: extraversion, agreeableness, conscientiousness, neuroticism, and openness [42]. It includes 10 items – two items per each dimension. Each item was scored using a five-point Likert-type scale, ranging from "strongly disagree” (= 1) and “strongly agree” (= 5), and the scores for each of the 10 items are summed to give a total score ranging from 2 to 10 on each dimension (2 items × 5 points). A higher score on any of the subdimensions indicates a greater presence of the characteristic trait of the participant's personality.

### Statistics

#### Sample size calculation

For exploratory studies, according to the Central Limit Theorem, a sample of at least 30 participants is required to obtain a Gaussian distribution [43]. Given the size of the population at the HEM-GE, a sample size of 100 participants was targeted.

#### Descriptive statistics

Data was first represented for the entire group, then for subgroups with and without PRMDs. Frequencies and percentages were calculated as categorical variables. Continuous variables were reported using means and standard deviations. The sub-groups were compared for each outcome with a chi-square test (categorical data) or t-test for unpaired groups (continuous data).

#### Network analysis

Network analysis aims to understand relationships and interactions between multiple variables. In mental health, it identifies how disorder symptoms are interconnected, determining central symptoms and uncovering patterns in a holistic, data-driven approach. For example, network analysis has shown that depression, stress, anxiety, and fear form a closely linked distress symptom network [44]. Most interestingly, it provides a synthesized graphical visualization where variables (e.g., “pain” or “stress”) appear as nodes, and associations are represented by edges, with thickness indicating the strength of correlations. Depending on the type of variables tested, the correlations used were adapted, for examples: Pearson correlations (for two continuous variables), point-biserial correlations (for continuous vs. binary variables). However, such visualizations can be misleading: nodes close together aren't necessarily highly related, and distant nodes aren't necessarily less related [45]. Network analyses offer several advantages over traditional methods such as regression analysis: they are applicable to various types of multivariate data, including cross-sectional, longitudinal, and time-series data, capturing the dynamic nature of psychological disorders by showing how symptoms change and interact over time. They allow grouping of related variables into "latent variables," enhancing theoretical understanding. This method informs diagnosis, treatment, and prevention strategies by refining diagnostic criteria, potentially leading to more accurate and nuanced classifications of psychological disorders.

In this study, the network analyses were conducted to investigate the overall correlational patterns within the dataset, aiming to better understand the associated factors, pain, and instrumental practice factors associated with PRMDs. If several closely associated variables are found to be grouped into a "latent variable", it may shed new light on the origins of PRMDs, for instance if this latent variable represents an underlying construct that may be associated with underlying mechanisms of PRMDs. Three networks were estimated via Gaussian Graphical Models (GGM):

- 1/ including all participants and all dependent variables associated with the PRMDs factor in the last 12 months (responding yes or no);
- 2/ including the participants presenting PRMDs over the last 12 months (responding yes) and all dependent variables related to pain intensity;
- 3/ including the participants presenting PRMDs over the last 12 months and all dependent variables related to pain interference.

On the graphs, only significant correlations are shown, Bonferroni corrections were conducted [46]. Green edges indicate positive correlations and red edges indicate negative correlations. A Bonferroni corrected *p*-value lower than 0.05 for the correlations was considered significant. All statistics including network models were estimated using the qgraph package of R (Version 1.4.4, 2012) within the R software environment [47].

## Results

### Participants

Two hundred and sixty-eight student musicians agreed to participate in the study, and 235 completed all questionnaires (mean age: 24.8 ± 4.3 years – range 18 – 42, Table 1). All music departments (instruments and voice) were represented, with a predominance of violin and piano. Participants spanned all five years of the bachelor and master curriculum. Students had been playing their main instrument or training their voice for an average of 15.5 ± 4.8 years. The number of training hours per day was estimated at 4.1 ± 1.7h. Preferred practice times were mornings (48% of students) and early afternoons (46%), and the main constraint cited was the scheduling of theory classes (59%) interfering with these preferred practice hours. 40% of students did not warm up or cool down before or after their instrumental practice.

**Table 1:** Participants’ demographics and music practice habits

	**Entire sample (*N* = 235)**	**PRMDs 12 months sample (*N* = 86)**	**Non-PRMDs sample (*N* = 149)**	***p value*s**
**Age** (years, Mean ± SD)	24.8 ± 4.3	24.0 ± 4.1	25.2 ± 4.5	0.04
**Sex** N (%)				
**Women**	151 (64%)	59 (69%)	92 (62%)	0.36
**Men**	84 (36%)	27 (31%)	57 (38%)	
**Height** (cm, Mean ± SD)	169 ± 9	168 ± 9	170 ± 9	0.06
**Weight** (kg, Mean ± SD)	63 ± 12	62 ± 13	64 ± 12	0.51
**Hand dominance** N (%)				
**Right**	209 (89%);	76 (88%);	133 (89%);	0.44
**Left**	21 (9%);	10 (12%);	11 (7%);	0.14
** Ambidextrous**	5 (2%)	0 (0%)	5 (3%)	
**Musical practice onset** (age, Mean ± SD)	8.1 ± 4.2	7.4 ± 4.2	8.5 ± 4.2	0.07
**Number of practice years on the main instrument** (years, Mean ± SD)	15.5 ± 4.8	15.6 ± 4.8	15.4 ± 4.8	0.66
**Second instrument** (yes) N (%)	142 (60%)	51 (59%)	91 (61%)	0.90
**Third Instrument (**yes) N (%)	40 (17%)	16 (19%)	24 (17%)	0.77
**Hours practiced played before starting at the HEM** (h/day, Mean ± SD)	3.5 ± 1.8	3.4 ± 1.6	3.5 ± 1.9	0.65
**Academic level** N (%)				
- **Bachelor 1**	49 (21%)	20 (23%)	29 (19%)	0.59
- **Bachelor 2**	40 (17%)	18 (21%)	22 (15%	
- **Bachelor 3**	31 (13%)	9 (10%)	22 (15%)	
- **Master 1**	51 (22%)	18 (21%)	33 (22%)	
- **Master 2**	64 (27%)	21 (25%)	43 (29%)	
**Hours practiced at the HEM** (h/day, Mean ± SD)	4.1 ± 1.7	4.1 ± 1.5	4.1 ± 1.8	0.93
**Breaks** N (%)				
- < **1h**	33 (14%)	17 (20%)	16 (11%)	***0.04***
- ≥ **1h**	101 (43%)	40 (47%)	61 (41%)	
- **Irregular**	101 (43%)	29 (34%)	72 (48%)	
**Preferred practice hours** N (%)				
- **Early AM**	113 (48%)			
- **End AM**	91 (39%)			
- **Early PM**	108 (46%)	-	-	-
- **End PM**	67 (28%)			
- **Evening**	39 (17%)			
**Musical training constraints** N (%)				
- **None**	59 (25%)	15 (17%)	44 (30%)	0.06
- **Neighbors**	22 (9%)	12 (14%)	10 (7%)	0.11
- **Student schedule**	138 (59%)	58 (67%)	80 (54%)	0.054
- **Work outside study**	58 (25%)	27 (31%)	31 (21%)	0.10
- **Deadlines**	51 (22%)	18 (21%)	33 (22%)	0.95
- **Available room or workplace**	71 (30%)	31 (36%)	40 (27%)	0.18
- **Other**	13 (6%)	7 (8%)	6 (4%)	0.30
**Physical exercises** N (%):				
- **Warm-up**	99 (42%)	29 (34%)	70 (47%)	***0.02***
- **Warm-up and recovery**	36 (15%)	21 (24%)	15 (10%)	
- **Recovery**	7 (3%)	3 (3%)	4 (3%)	
- **None**	93 (40%)	33 (38%)	60 (40%)	
**Musical practice position** N (%):				
- **Sitting**	78 (33%)	27 (31%)	51 (34%)	0.36
- **Standing**	50 (21%)	15 (17%)	35 (23%)	
- **Both**	107 (46%)	44 (51%)	63 (42%)	
**Practice posture upper limbs (*N* = 235)** N (%)				
- **Frontal, neutral, singing**	123 (52%)	41 (48%)	82 (55%)	0.15
- **One left, one right, quad left**	96 (41%)	42 (49%)	54 (36%)	
- **Others**	16 (7%)	3 (2%)	13 (9%)	

In the last column, *p*-values resulting from the Chi^2^/t-tests comparing PRMDs and Non-PRMDs samples are provided

48% of students worked beside their study to support themselves (Table 2). Only 40% engaged in regular PA (> 150min per week). Average sleep duration was 7.1 ± 0.9h and 65% reported sleep duration ≤ 7h.

**Table 2:** Lifestyle characteristics

	**Entire sample** **(*N* = 235)**	**PRMDs 12 months** **sample** **(*N* = 86)**	**Non-PRMDs sample (*N* = 149)**	***p value*s**
**Student job (yes)** N (%)	113 (48%)	45 (52%)	68 (46%)	0.39
**Regular meals (yes)** N (%)	200 (85%)	76 (88%)	124 (83%)	0.38
**Balanced diet (yes) N (%)**	180 (77%)	69 (80%)	111 (74%)	0.40
**Sleep duration**				
- **Hours/night** (Mean ± SD)	7.1 ± 0.9	7.1 ± 0.9	7.2 ± 0.8	0.50
- ≤ **7h** N (%)	153 (65%)	56 (65%)	97 (65%)	1.00
- ≥ **8h** N (%)	82 (35%)	30 (35%)	52 (35%)	1.00
**Sleep quality (scale / 5)** Mean ± SD	2.8 ± 1.3	3.0 ± 1.4	2.7 ± 1.2	0.056
**Addiction** N (%)	29 (12%)	11 (13%)	18 (12%)	0.73
**Physical activity – WHO recommendations** N (%)** (*n* = 217)**				
- **Yes**	86 (40%)	30 (39%)	56 (40%)	0.99
- **No**	131 (60%)	47 (61%)	84 (60%)	

In the last column, *p*-values resulting from the Chi^2^/t-tests comparing PRMDs and Non-PRMDs samples are provided

### Clinical characteristics

Fifty-seven percent of students reported suffering from PRMDs, of which only 23% received a medical diagnosis. 37% had experienced PRMDs over the last 12 months and 13% in the last 7 days (Table 3). The students with PRMDs had a Numeric Rating Scale (NRS): 3.8 ± 2.6/10) for pain interference with musical practice and a NRS: 2.8 ± 1.7/10 for Pain intensity.

**Table 3:** Clinical characteristics of the participants for validated questionnaires

	**Entire sample (*N* = 235)**	**PRMDs 12 months sample (*N* = 86)**	**Non-PRMDs sample (*N* = 149)**	***p value*s**
***Primary outcome***				
**PRMDs: frequency** N (%)				
- **In the past**	133 (57%)	86 (100%)	47 (32%)	0.001
- **Last 12 months**	86 (37%)	86 (100%)	N/A	
- **Last 30 days**	44 (19%)	43 (50%)	N/A	
- **Last week**	30 (13%)	28 (33%)	N/A	
- **Pain interference (/10)** Mean ± SD	1.5 ± 2.5	3.8 ± 2.6	N/A	
- **Pain intensity (/10)** Mean ± SD	1.1 ± 1.8	2.8 ± 1.7	N/A	
***Secondary outcomes***				
**Str/K10** N (%)				
- **Well**	60 (26%)	12 (14%)	48 (32%)	0.001
- **Mild mental disorders**	55 (23%)	16 (19%)	39 (26%)	
- **Moderate mental disorders**	56 (24%)	26 (30%)	30 (20%)	
- **Severe mental disorders**	64 (27%)	32 (37%)	32 (21%)	
**MPA** N (%)				
- **No MPA**	2 (0.9%)	1 (1%)	1 (1%)	0.09
- **Low MPA**	42 (18%)	8 (9%)	34 (23%)	
- **Average MPA**	78 (33%)	28 (33%)	50 (33%) (34%)	
- **Above average MPA**	87 (37%)	37 (43%)	50 (33%) (34%)	
- **High MPA**	25 (11%)	11 (13%)	14 (9%)	
- **Extremely high MPA**	1 (0.4%)	1 (1%)	0 (0%)	
**Perfectionism** Mean ± SD				
- **Total score**	109.9 ± 23.4	115.3 ± 22.7	106.8 ± 23.4	0.007
- **Explicit**	37.7 ± 9.0	39.3 ± 8.5	36.7 ± 9.2	0.03
- **Implicit**	72.2 ± 19.4	76 ± 19	70.1 ± 19.4	0.02
**Frequency** N (%)				
- **Low**	79 (34%)	26 (30%)	53 (36%)	0.004
- **Medium**	80 (34%)	21 (24%)	59 (40%)	
- **High**	76 (32%)	39 (45%)	37 (25%)	
**Fatigue** Mean ± SD				
- **Bimodal with QMem**	4.0 ± 3.3	4.8 ± 3.4	3.5 ± 3.1	0.005
- **Bimodal without QMem**	3.9 ± 3.1	4.6 ± 3.3	3.4 ± 3	0.006
- **Likert with QMem**	14.8 ± 5.7	16.2 ± 6.3	14 ± 5.1	0.005
- **Likert without QMem**	13.7 ± 5.5	15.1 ± 6.2	12.9 ± 4.9	0.006
**Self-rated health (score /4)** Mean ± SD				
- **Item 1**	2.4 ± 0.6	2.3 ± 0.6	2.5 ± 0.6	0.01
- **Item 2**	2.0 ± 0.8	1.8 ± 0.8	2.1 ± 0.8	0.02
- **Item 3**	2.1 ± 0.6	2.0 ± 0.6	2.1 ± 0.6	0.04
- **Item 4**	2.5 ± 0.6	2.3 ± 0.7	2.6 ± 0.6	0.003
- **Mean**	2.2 ± 0.5	2.0 ± 0.5	2.2 ± 0.5	0.003

In the last column, *p*-values resulting from the Chi^2^/t-tests comparing PRMDs and Non-PRMDs samples are provided. *PRMDs* playing-related musculoskeletal disorders, *K10* Kessler Psychological Distress Scale, *QMem* question memory, *MPA* musical performance anxiety

For the STR/K10 results, 51% of students were classified as having moderate or severe levels of psychological distress. 48% reported an above average, high or extremely high-performance stress in the K-MPAI-R results. The results of perfectionism, fatigue, SRH are represented in Table 3.

### Comparisons between PRMDs and non-PRMDs subgroups

Comparison between the PRMDs group and the non-PRMDs groups revealed a significant increase in pathological history (*p* < 0.001), psychological distress (Str/K10) (*p* < 0.001), perfectionism (*p* < 0.007) and fatigue (*p* < 0.005) in the presence of PRMDs *(*Table 3*)*. No significant differences were observed between groups for physical factors (posture and PA), except for warm-up (*p* < 0.020, Table 1).

### Network analyses

**Fig. 1:**
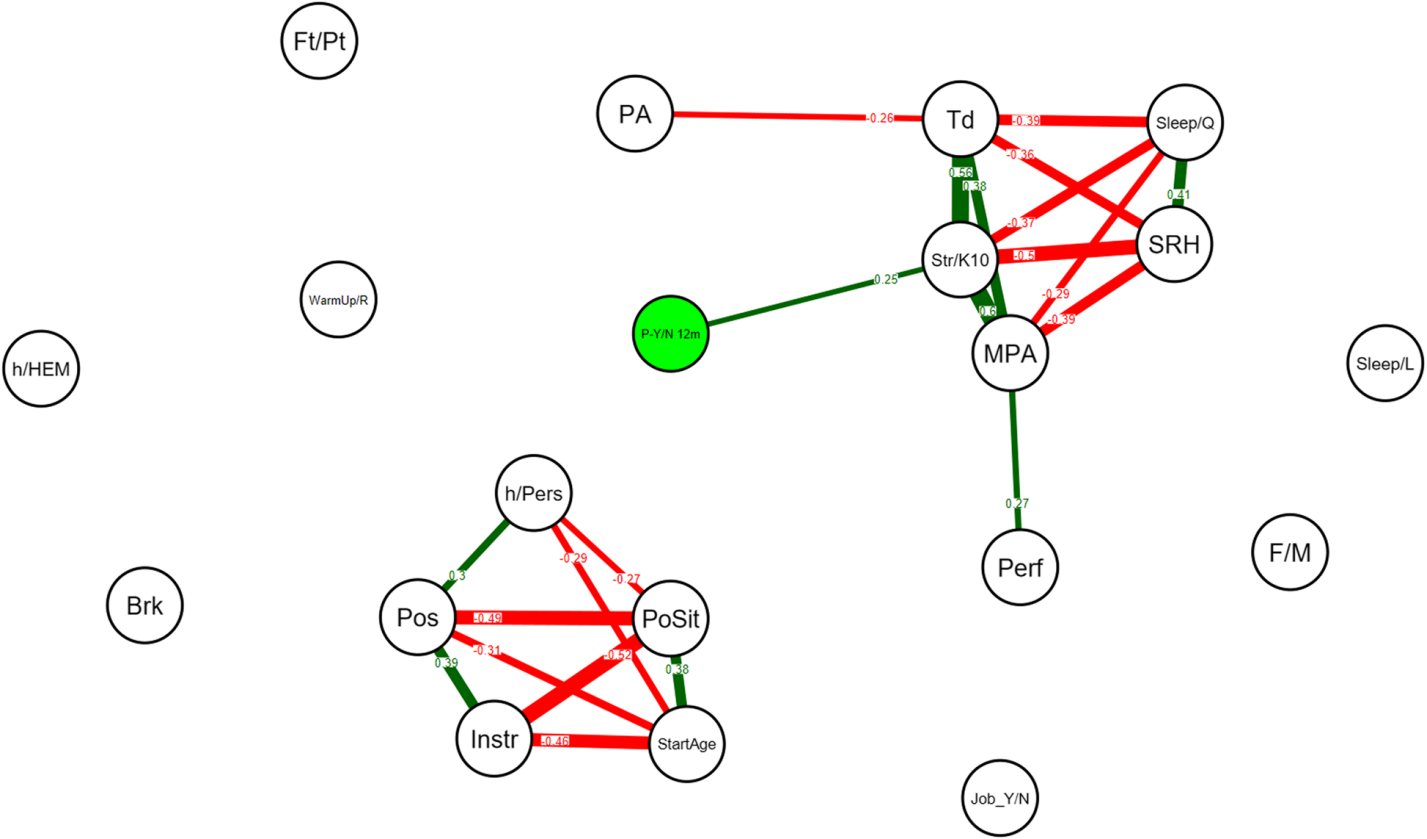
Network analysis on all variables across all participants (*N* = 235), on absence (no) or presence (yes) of the PRMDs variable over the last 12 months. A line between the nodes representing the variables indicates a significant correlation. Absence of a line (edge) between two variables indicates a non-significant correlation. Green edges indicate positive correlations and red edges indicate negative correlations. The number on the line (correlation coefficient) and its thickness represent the strength of the correlation. Only significant correlations (*p* < 0.05) are shown, using Bonferroni corrections. P-Y/N 12m: Pain yes – no in the last 12 months (MPIIQM), Str/K10: Kessler Psychological Distress Scale, MPA: Kenny Music Performance Anxiety Inventory, Td: Tiredness, SRH: Self-rated Health, PA: Physical Activity, Sleep/Q: sleep quality, Perf: perfectionism, Pls: Pleasantness, Sleep/L: Sleep duration, F/M: gender, h/Pers: weekly hours played personally, PoSit: posture sitting or standing, Pos: posture according with upper limb position, StartAge: Starting Age Playing Instrument, Instr: instrument, Brk: break’ organization, Neuro: neurological previous disease, h/HEM: Weekly hours played at HEM-GE,, Ft/Pt: Full or Part-time job, Job_Y/N: student job,, WarmUp/R: warm-up and recovery exercises

Network analysis revealed a moderate association between PRMDs over the last 12 months and Str/K10 values *(r* = 0.25). The Str/K10 were moderate to strongly associated with MPA *(r* = 0.60), fatigue *(r* = 0.56), SRH *(r* = -0.5) and sleep quality *(r* = -0.37) (Fig. 1). PA was not associated with the Str/K10 value but with fatigue (*r* = -0.26).

**Fig. 2:**
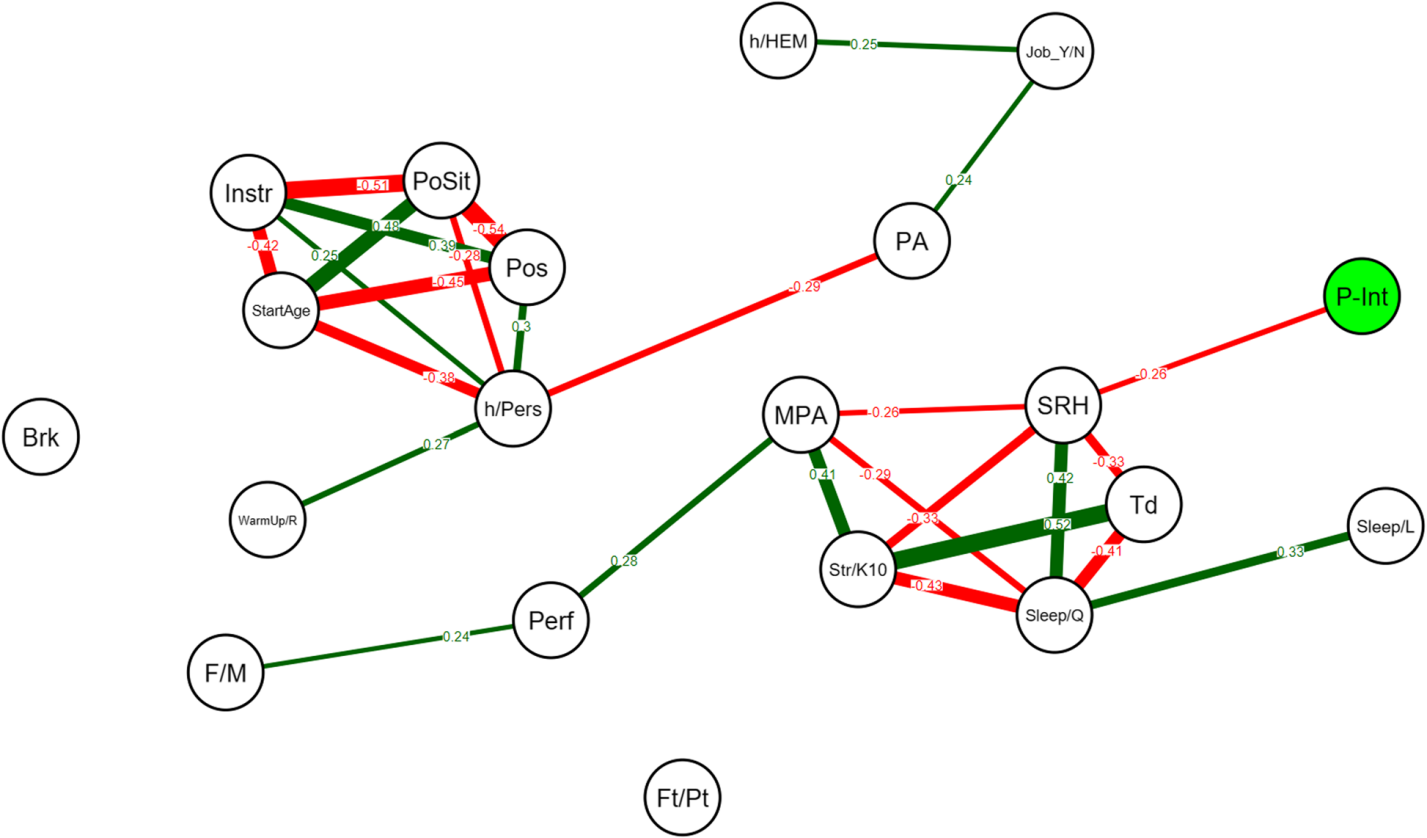
Network analysis on the group of participants presenting PRMDs over the last 12 months (*N* = 86) including pain intensity and all dependent variables. A line (edge) between the nodes representing the variables indicates a significant correlation. Absence of a line (edge) between two variables indicates a non-significant correlation. Green edges indicate positive correlations and red edges indicate negative correlations. The number on the line (correlation coefficient) and its thickness represent the strength of the correlation. Only significant correlations (*p* < 0.05) are shown, using Bonferroni corrections. P-Int: pain intensity, SRH: Self-rated Health, Str/K10: Kessler Psychological Distress Scale, MPA: Kenny Music Performance Anxiety Inventory, Td: Tiredness, PA: Physical Activity, Sleep/Q: sleep quality, Perf: perfectionism, Pls: Pleasantness, Sleep/L: Sleep duration, F/M: gender, h/Pers: weekly hours played personally, PoSit: posture sitting or standing, Pos: posture according with upper limb position, StartAge: Starting Age Playing Instrument, Instr: instrument, Brk: break’ organization, Neuro: neurological previous disease, h/HEM: Weekly hours played at HEM-GE, Extra:, Ft/Pt: Full or Part-time job, Job_Y/N: student job, WarmUp/R: warm-up and recovery exercises

When the network analysis exclusively focused on the participants presenting PRMDs over the last 12 months (*N* = 86), pain intensity (P-int) was negatively correlated with SRH values and the SRH value was mainly associated with sleep quality (*r* = 0.42), fatigue *(r* = -0.33), Str/K10 *(r* = -0.33) and MPA *(r* = -0.26) values (Fig. 2).

**Fig. 3:**
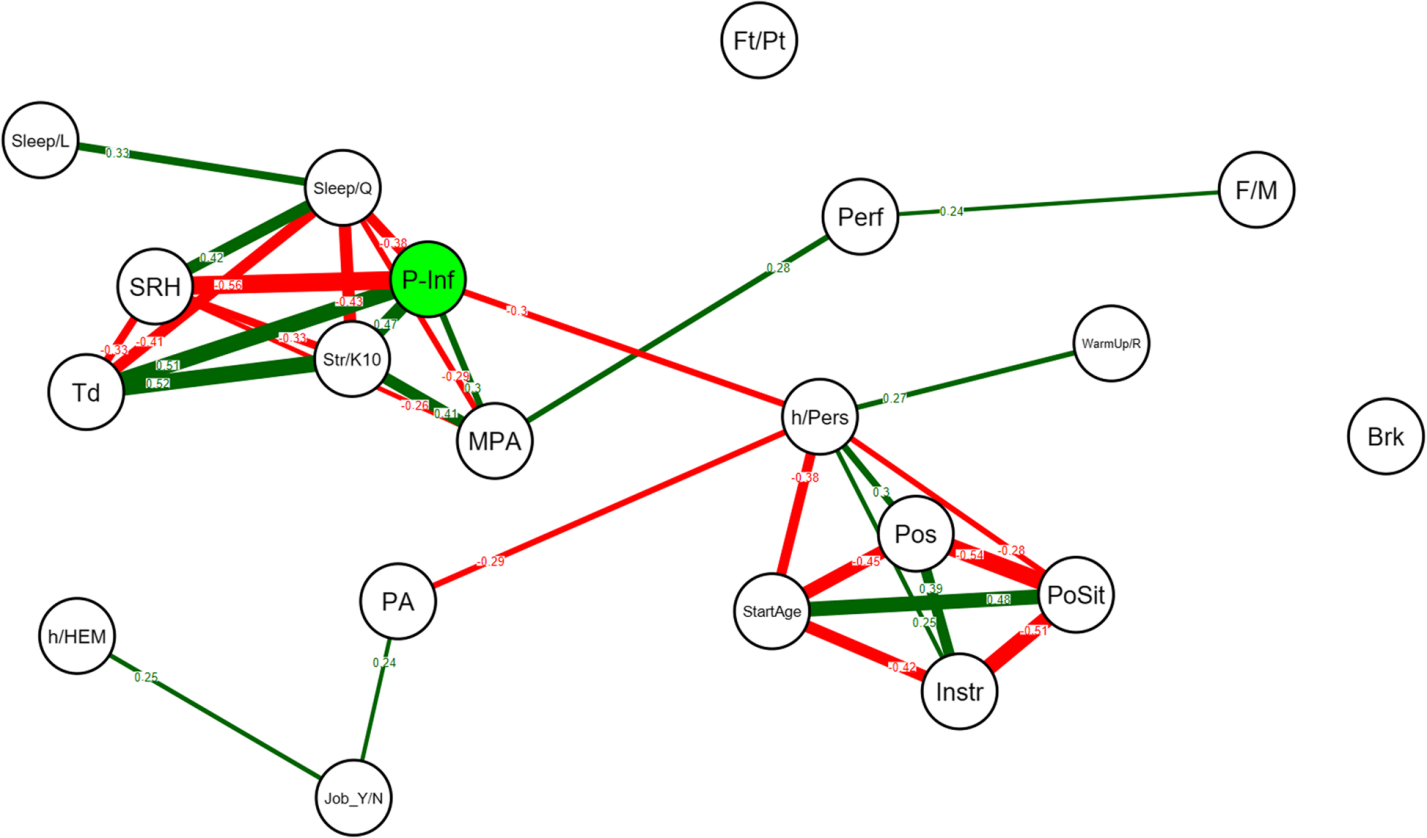
Network analysis on the group of participants presenting PRMDs over the last 12 months (*N* = 86) including pain interference and all dependent variables. A line between the nodes represents a significant correlation. Red represents a negative correlation, while green represents a positive correlation. The number on the line and its thickness represent the strength of the correlation. Only significant correlations (*p* < 0.05) are shown, using Bonferroni corrections. P-Inf: pain interference, SRH: Self-rated Health, Str/K10: Kessler Psychological Distress Scale, MPA: Kenny Music Performance Anxiety Inventory, Td: Tiredness, PA: Physical Activity, Sleep/Q: sleep quality, Perf: perfectionism, Pls: Pleasantness, Sleep/L: Sleep duration, F/M: gender, h/Pers: weekly hours played personally, PoSit: posture sitting or standing, Pos: posture according with upper limb position, StartAge: Starting Age Playing Instrument, Instr: instrument, Brk: break’ organization, Neuro: neurological previous disease, h/HEM: Weekly hours played at HEM-GE, Extra:, Ft/Pt: Full or Part-time job, Job_Y/N: student job, WarmUp/R: warm-up and recovery exercises

In the PRMDs group, pain interference with musical practice (P-Inf) was strongly negatively associated to SRH *(r* = -0.56), positively associated with fatigue *(r* = 0.51), Str/K10 *(r* = 0.47), MPA *(r* = 0.30) and negatively associated to sleep quality *(r* = -0.38), Fig. 3) and the MPA was linked to perfectionism *(r* = 0.28).

## Discussion

### Main results: psychological health is the key factor related to PRMDs

Network analysis highlighted that psychological distress was the sole factor directly associated with the presence or absence of PRMDs, rather than the number of hours played or work hygiene (including PA, warm-up, recovery exercises and posture). For participants with PRMDs, this same factor is also linked to pain interference with musical practice. These findings reinforce earlier studies that demonstrate a strong connection between stress factors and musculoskeletal pain in both pre-professional and professional musicians [21, 22]. Although our cross-sectional study does not permit conclusions about causation between stress and PRMDs, psychological vulnerability appears to be a critical issue among musicians [8, 19], including young student musicians.

Surprisingly, evidence suggests that playing skill level does not significantly impact the occurrence or severity of PRMDs. The large-scale RISMUS study on 997 music students attribute PRMDs prevalence to factors like intensive practice and limited rest, rather than skill level [17]. Similarly, Baadjou et al. 2016, 2018 [20, 48] identify physical strain, instrument demands, and stress as primary contributors, with no reduction in PRMDs among advanced players despite health-focused interventions. Joyce et al. 2024 [49] also underscores demographic factors over skill level in PRMDs incidence. Research indicates that music students face elevated risks of stress and depression compared to peers in other higher education fields [5, 6, 14], except for students involved in high-level sports activities [28]. The elevated participation rate in the current study underscores the importance student musicians attach to health issues.

Compared to Cruder et al. 2020 [17], our study reported a lower prevalence of PRMDs over the past year, but a higher Str/K10 score. Psychological health proved to be a critical issue for student musicians, with over 50% experiencing moderate to severe psychological distress, and nearly 50% reporting above-average to high levels of MPA. Both studies identified psychological factors, particularly stress and MPA, as significant contributors to PRMDs. However, Cruder et al. 2020 also found physical factors to be relevant [17]. This discrepancy could be due to methodological approaches. Our network analysis explored the interrelationships between physical and psychological factors, offering a deeper understanding of the relationships between all variables in the context of PRMDs. This advanced multivariate method might offer new perspectives addressing the intertwined nature of mental and physical health in this population,​​ and ultimately lead to optimal intervention programs. Some variables were indirectly linked to PRMDs through their association with Str/K10 scores in students with PRMDs. This creates a vicious cycle where pain increases psychological distress, which then worsens self-rated health, fatigue, sleep quality, and MPA. Increased fatigue then disrupts posture, coordination, proprioception, and muscle tension, all contributing to PRMDs [24, 29]. Thus, managing sleep quality, MPA and fatigue (including both psychological and physical aspects), could help prevent both physical and psychological strain [8, 50].

### Stress at the heart of PRMDs

Interestingly, the results highlighted that the presence of PRMDs is more strongly correlated with the Str/K10 score than the K-MPAI-R score. These results raise questions about whether individuals with higher psychological sensitivity or distress are initially more likely to pursue music studies [10]. In our study, student musicians with PRMDs exhibited higher Str/K10 scores and increased perfectionism than students without PRMDs, but the groups showed no differences in the BFI-10 for personality traits. Unlike fields such as medicine or law, choosing to study music is primarily driven by intrinsic motivation and passion, rather than the pursuit of high social status [28]. Despite this, performance excellence remains the ultimate goal. This intrinsic motivation and drive for excellence may explain the strong link between pain and distress, as pain profoundly impacts musicians’ performance and thus their professional future. For them, the ability to perform well, threatened by pain, is central to their identity and impacts their entire life sphere, including social relationships [10]. Mild to moderate pain, which does not significantly interfere with practice, often leads musicians to adopt a "no pain, no gain" mentality, delaying early diagnosis [6, 51]. Conversely, intense pain that interferes with practicing and performing strongly impacts psychological health and interferes with musical practice and learning processes, leading to doubts and anxiety about the future [10, 28]. Our results reveal a vicious cycle, with high correlations between pain interference, Str/K10 scores, MPA, fatigue, sleep quality, and SRH. Intense pain eventually drives student musicians to seek professional help, often too late, especially since suitable professionals are scarce [10]. Early treatment is also hindered by the varied attitudes of music teachers, who may ignore health issues or attribute pain solely to poor technique [19].

However, when intense pain has already set in, it is not easily overcome. This is partly because the brain has learned to associate music practice with pain through a plastic reorganization of functional brain activity [52]. Moreover, in musicians, motor learning for non-optimally performed movements, especially those practiced under stress and anxiety, is encoded in the brain just like optimally performed movements [53]. The brain does not differentiate between the quality of movements during the encoding process; rather, it strengthens whichever patterns are repeatedly practiced [54]. Our study results about PRMDs and psychological health underscore the necessity for preventive approaches to integrate health management from the very start of professional music studies. Implementing these strategies within the music curriculum can help mitigate the adverse effects of pain and enhance students’ performance and well-being [10].

### Psychological profiles and PRMDs

Students with PRMDs had higher Str/K10 scores and higher perfectionism but showed no differences in the personality traits. Initially, we anticipated that, for instance, neuroticism would impact the occurrence of PRMDs. A likely explanation is that the short form of the BFI-10 does not capture sufficient detail to observe subtle personality differences that might impact PRMDs, explaining the contrast to past research indicating that personality traits such as neuroticism are linked to stress and pain. For instance, a positive association between neuroticism and job burnout was reported [55]. However, other factors like psychological distress and perfectionism may play a more direct role in PRMDs in the specific context​ of professional music practice and performance. Both medical and music students are perfectionist; this tendency is stronger in early study years and linked to anxiety [56].

### Fatigue, a potential connector between psychological and physical health

While fatigue is often identified as a key risk factor for injury in musicians [50], Cruder et al. 2020 [17] only showed an association with general musculoskeletal disorders, not directly with PRMDs as observed in our study. However, in the current exploratory study, it is particularly interesting to observe an association between general fatigue and psychological distress, a factor that is very strongly associated with PRMDs. Thus, the Chalder scale, which includes both physical and psychological fatigue [57], seems a particularly relevant self-questionnaire for exploring and understanding the close links between physical and psychological disorders in student musicians. McCrary et al. 2022 [50] recently proposed a fatigue self-assessment for musicians based on workload and physical and psychological stressors, as recommended in sports for injury prevention. Furthermore, Möller et al. 2018 [24] observed that fatigue induces altered electromyographic activity in those with PRMDs, potentially linked to early physical fatigue in musicians facing high mental demands in a competitive environment. Psychological fatigue also appears to be greater at the beginning of study at university levels [27], highlighting the importance of better exploring this variable in order to propose appropriate fatigue management [50].

### The role of physical factors

According to the literature, PA seems an excellent way to combat stress among students [7]. However, the current study found that 60% of students did not meet WHO recommendations [58]. We used the IPAQ-SF and the threshold of 150 min/week of moderate to vigorous PA according to the 2020 WHO recommendations. This questionnaire may not be suitable for musicians in the context of PRMDs as it only measures the duration and intensity of activity, not its specific nature. It would be interesting to distinguish between sedentary behavior and relative inactivity (e.g., musicians who play seated) and also to investigate the specific content of the PA practiced by music students. Not all PA affect stress equally, some might be more protective of psychological health and meanwhile also improve general physical capacities useful to musicians [59]. Optimal PA for musicians may vary also as a function of the instrument played, and the anatomical localization of PRMDs. For instance, the IPAQ-SF does not inform on the involvement of the upper part of the body, where most pain experiences manifest.

Contrary to our expectations that physical factors like posture would be associated with PRMDs [17], the network analysis used here found no link between physical factors and PRMDs. Notwithstanding, the comparison between students with and without PRMDs showed that musicians without PRMDs take more irregular breaks and are more likely to practice warm-up and recovery exercises, indicating better physical awareness and practice organization. This successful strategy provides precious information on how to prevent pain. However, in PRMDs follow-ups by health professionals, work habits, fatigue management, and organization are rarely considered. In contrast, organizing work by content, intensity, and breaks is widely practiced in sports and, more recently, among dance artists [60]. These approaches, often unknown to musicians, are particularly relevant for better controlling physical and mental fatigue throughout the year.

### Transition to higher education: a key moment for preventive advice

The transition to higher education in music performance is risky; however, the year of study was not a risk factor associated with pain. Descriptive questions in the MPIIQM revealed that practice hours varied little the year before and after entering higher music education. The period before entry, when aspiring musicians likely work very hard to pass entrance exams, indicates that the increase in work intensity occurs earlier. This justifies the need to address health issues and associated factors already in preparatory classes for higher education in music performance.

### Practical implications: improving health monitoring for music students

In line with the Guptill’s [10] and Detari models [61] and our results, the prevention advices should be based on the interplay between physical, psychological and social factors that influence the health of musicians. These models highlight the importance of a holistic approach to health promotion, integrating multiple aspects of a musician's life and environment. Musicians play a crucial role in managing their own health, given the demanding nature of their practice [62–64], while half the music students reported that they participated in preventive health behavior during university education [65]. Thus, a person-centered approach enhances practical health knowledge transfer and preventive action effectiveness [15]. To support this, health education should be an integral part of music training [66], especially as these approaches seem to improve students' health [25]. However, it is essential to develop and clearly define the roles of both music teachers and students in health prevention and promotion.

According to Guptill et al. 2011, health promotion relies on three main areas where student musicians themselves can play a central role: health knowledge, good daily practices, and lifestyle habits [10], as well as the early detection of risk factors and health problems using Patient-Reported Outcome Measures (PROMs) [12, 63, 67]. Musicians should also actively improve their daily well-being and behaviour [61, 68]. Our results indicate that these approaches must address both physical and psychological health, with a specific focus on stress management, both generally and specifically during performance. Distancing from work is suggested as a potential stress reducer, with leisure activities helping to prevent excessive focus on practicing one's primary instrument [4].

For health promotion to be effective in a pedagogical context, music teachers must also be involved. Providing teachers with musicophysiology instruction could positively impact their teaching style [69]. Their primary prevention roles include improving practical health knowledge, integrating warm-up and recovery routines, identifying risk factors, providing appropriate advice [69], and fostering a positive work environment that respects physical, psychological and social health [19, 51]. Notably, a teaching approach exclusively focused on error prevention can generate anxiety and negatively affect psychological health. Excessive focus on error correction and technical precision can shift energy away from enhancing sound quality and musical expression, potentially hindering motor learning pathways instead of strengthening them [13]. Additionally, teachers should avoid giving advice based on personal experience, as all students are unique [51]. An important point is that expectations should be adapted to the level of the musician and should be realistic in order to facilitate moments of satisfaction during performance. This positive feeling could help to reduce stress and muscular tension.

### Limitations

The study has some limitations. First, the sample size that is relatively restraint and only concerns the HES-GE population. However, the 43% response rate well represents the population, allowing the study of the specific context of the HEM-GE, with aiding in the implementation of specific preventive actions. Potentially, students with PRMDs responded more frequently, thus overestimating health issues. However, the high response rate and consistent results with other studies suggest this is unlikely. Despite using validated questionnaires, self-reporting may have caused misconceptions, such as for PA levels. Nonetheless, self-appraisal ensures anonymity and reflects how students perceive their own health and associated factors. Finally, the results showed a high level of psychological distress (Str/K10). It might therefore have been appropriate to exclude these students. However, many (student) musicians suffer from these disorders and to exclude them when they are considered fit to follow the normal curriculum and practice their main instrument would have severely limited these exploratory results by not taking into account this category of students. Furthermore, the results obtained are close to those of the Cruder et al. 2020 study [17], which seems to confirm the high prevalence of these disorders in music performance studies.

## Conclusion

This exploratory study revealed the critical correlation between psychological distress and MPA for PRMDs among student musicians. It highlights the need for integrated health promotion strategies that address both physical and mental health, with a focus on psychological health support, and early tailored interventions. Incorporating structured health education and supportive environments in music curricula can reduce PRMDs prevalence and improve student well-being. Further research should explore the impact of different physical activities and the effectiveness of preventive strategies. Integrating mental health support in music education is essential for student musicians' well-being and performance.

## Supplementary Information

Supplementary Material 1.

## Data Availability

The data that support the findings of this study are available from the corresponding author [AVB], upon reasonable request.

## Abbreviations

BFI-10: Big Five Inventory
IPAQ-SF: International Physical Activity Questionnaire-Short Form
K-MPAI-R: Kenny Music Performance Anxiety Inventory
MET: Metabolic Equivalent Task
MPIIQM: Musculoskeletal Pain Intensity and Interference Questionnaire for Musicians
MPA: Music Performance anxiety
NRS: Numeric Rating Scale
PA: Physical Activity
PMQ: Perfectionism Motivation Questionnaire
PRMDs: Playing-Related Musculoskeletal Disorders
Str/K10: Kessler Psychological Distress Scale
WHO: World Health Organisation
