# Supplementary material for: Network analyses of physical and psychological factors of playing-related musculoskeletal disorders in student musicians: a cross-sectional study
Source: BMC Musculoskelet Disord. 2024 Nov 30;25:979. doi: 10.1186/s12891-024-08103-8 (PMC11607880; doi:10.1186/s12891-024-08103-8)
Supplement: Supplementary file 1 — Supplementary Material 1. [file 12891_2024_8103_MOESM1_ESM.pdf]

Pain assessment in musicians

Page 1/9

Your first and last name were requested for the consent (previous page). However, all the answers to the following questions are completely anonymous and it will not be possible to identify you.

Hello, Thank you for your participation. This research conducted by the Haute Ecole de Santé in collaboration with the Haute Ecole de Musique of Geneva - Neuchâtel aims to evaluate the health of student musicians.
The questions concern various aspects of your life such as your musical practice, your general feelings, your physical activity...
There are no right or wrong answers; we simply ask you to answer each question as spontaneously as possible.
It is best to complete the entire survey in one go if you have the opportunity. Otherwise, do not interrupt your participation during the survey, but only after clicking < < Submit >> at the end of a page. Submitting locks the answer to the questions on a page, so it is no longer possible to go back after changing pages. A full participation takes about 20-40 minutes. Inclusive writing has not been used to facilitate readability.
To thank you for your participation, you are entitled to a gift voucher worth CHF 30.00 to be used in a Payot bookstore. The vouchers will be distributed to all participants who completed all the questions at the end of the questionnaire data collection in late April / early May. Thank you for participating.

If the results of your answers suggest that there is a physical or psychological disorder, do you wish :

◯ not to be informed
◯ to be informed so that you can contact a health professional if you wish

---

What is your age?
{mpiiqm_q1} years

Gender

◯ Male ◯ Female

What is your main musical instrument at the HEM?

____
(Includes singing)

With respect to your studies, are you registered:

◯ Full time ◯ Part time

For how many years have you played your main instrument?

{mpiiqm_q5} years

For how many years and months did you study at a (under)graduate university of music?

{mpiiqm_q6_ans} year(s) and {mpiiqm_q6_mois} month(s)

If it is an exact number of years please choose 0 months

On average, how many hours per week do you spend playing your main instrument (or singing for the singers) during HEM supervised activities (this includes lessons, rehearsals, performances) ?

{mpiiqm_q7} hours

On average, how many hours per week do you spend playing your main instrument (or singing for singers) outside HEM supervised activities (this includes individual practice, chamber music, solo performances, demonstration when teaching, gigs, other)?

{mpiiqm_q8} hours

Playing-related (or singing-related for singers) musculoskeletal problems are defined as "pain, weakness, numbness, tingling, or other symptoms that interfere with your ability to play your instrument at the level to which you are accustomed". This definition does not include mild transient aches and pains.

Have you ever had pain/problems that have interfered with your ability to play your instrument at the level to which you are accustomed?

◯ Yes ◯ No

Have you ever had pain/problems that have interfered with your ability to play your instrument (or sing for singers) at the level to which you are accustomed during the last 12 months ?

◯ Yes ◯ No

Have you had pain/problems that have interfered with your ability to play your instrument (or sing for singers) at the level to which you are accustomed during the last month (4 weeks) ?

◯ Yes ◯ No

Currently (in the past 7 days), do you have pain/problems that interfere with your ability to play your instrument (or sing for singers) at the level to which you are accustomed?

◯ Yes ◯ No

The lists below enumerate the different front parts of the body.

Check off each area where you are experiencing pain/problems.

{schema_corps_av} Head: {tete}

Trunk: {tronc}

Upper extremity: {ms} Lower extremety: {mi}

The lists below enumerate the different back parts of the body.

Check off each area where you are experiencing pain/problems.

{schema_corps_ar} Head: {tete_a}

Trunk : {tronc_a}

Upper extremity: {ms_a} Lower extremety: {mi_a}

The drop-down menu below enumerates the different front AND back parts of the body.
Select the area that is the most painful.

◯ Mouth
◯ Front of the head
◯ Throat
◯ Front of the left shoulder
◯ Front of the right shoulder
◯ Front of the left shoulder
◯ Front of the right shoulder
◯ Fold of the left arm
◯ Fold of the right arm
◯ Front of the left forearm
◯ Front of the right forearm
◯ Front of the left wrist
◯ Front of the right wrist
◯ Front of the left hand palm
◯ Front of the right hand palm
◯ Left thumb
◯ Right thumb
◯ Front of other left fingers
◯ Front of other right fingers
◯ Chest
◯ Belly
◯ Front of the pelvis
◯ Front of the left thigh
◯ Front of the right thigh
◯ Left knee
◯ Right knee
◯ Front of the left leg
◯ Front of the right leg
◯ Front of the left ankle
◯ Front of the right ankle
◯ Front of the left foot
◯ Front of the right foot
◯ Back of the head
◯ Neck
◯ Back of the left shoulder
◯ Back of the right shoulder
◯ Back of the left arm
◯ Back of the right arm
◯ Left elbow
◯ Right elbow
◯ Back of the left forearm
◯ Back of the right forearm
◯ Back of the left wrist
◯ Back of the right wrist
◯ Back of the left hand
◯ Back of the right hand
◯ Upper back
◯ Middle of the back
◯ Lower back
◯ Back of the pelvis
◯ Back of the left thigh
◯ Back of the right thigh
◯ Hollow of the left knee
◯ Hollow of the right knee
◯ Back of the left leg
◯ Back of the right leg
◯ Back of the left ankle
◯ Back of the right ankle
◯ Back of the left foot
◯ Back of the right foot

The next four questions relate ONLY to PAIN. Please answer with reference to the ONE area that you selected in the previous question.

Please rate your pain by circling the one number that best describes your pain at its worst in the last week.
(0 = No pain & 10 = Pain as bad as you can imagine)

◯ 0 ◯ 1 ◯ 2 ◯ 3 ◯ 4 ◯ 5 ◯ 6 ◯ 7 ◯ 8 ◯ 9 ◯ 10

Please rate your pain by circling the one number that best describes your pain at its least in the last week.
(0 = No pain & 10 = Pain as bad as you can imagine)

◯ 0 ◯ 1 ◯ 2 ◯ 3 ◯ 4 ◯ 5 ◯ 6 ◯ 7 ◯ 8 ◯ 9 ◯ 10

Please rate your pain by circling the one number that best describes your pain on average in the last week.
(0 = No pain & 10 = Pain as bad as you can imagine)

◯ 0 ◯ 1 ◯ 2 ◯ 3 ◯ 4 ◯ 5 ◯ 6 ◯ 7 ◯ 8 ◯ 9 ◯ 10

Please rate your pain by circling the one number that tells how much pain you have right now.
(0 = No pain & 10 = Pain as bad as you can imagine)

◯ 0 ◯ 1 ◯ 2 ◯ 3 ◯ 4 ◯ 5 ◯ 6 ◯ 7 ◯ 8 ◯ 9 ◯ 10

The remainder of the survey relates to both PAIN and/or PROBLEMS.
For each of the following, circle the one number that describes how, during the past week, pain/problems have interfered with your:

Mood
(0 = Does not interfere & 10 = Completely interferes)

◯ 0 ◯ 1 ◯ 2 ◯ 3 ◯ 4 ◯ 5 ◯ 6 ◯ 7 ◯ 8 ◯ 9 ◯ 10

Enjoyment of life
(0 = Does not interfere & 10 = Completely interferes)

◯ 0 ◯ 1 ◯ 2 ◯ 3 ◯ 4 ◯ 5 ◯ 6 ◯ 7 ◯ 8 ◯ 9 ◯ 10

For each of the following, during the past week, as a result of your pain/problems, did you have any difficulty:

Using your usual technique for playing your instrument?
(0 = No difficulty & 10 = Unable)

◯ 0 ◯ 1 ◯ 2 ◯ 3 ◯ 4 ◯ 5 ◯ 6 ◯ 7 ◯ 8 ◯ 9 ◯ 10

Playing your musical instrument because of your symptoms?
(0 = No difficulty & 10 = Unable)

◯ 0 ◯ 1 ◯ 2 ◯ 3 ◯ 4 ◯ 5 ◯ 6 ◯ 7 ◯ 8 ◯ 9 ◯ 10

Playing your musical instrument as well as you would like?
(0 = No difficulty & 10 = Unable)

◯ 0 ◯ 1 ◯ 2 ◯ 3 ◯ 4 ◯ 5 ◯ 6 ◯ 7 ◯ 8 ◯ 9 ◯ 10

General questions

Page 2/9

Socio-demographic informations

Height: {ql1} cm

Weight: {ql2} kg

Do you write with your right or left hand?

◯ Right ◯ Left ◯ Ambidextrous

Do you have a student job?

◯ Yes ◯ No

Which one?

____

How many hours per week?

____

In the past 12 months, have you had a history of:

☐ Neurological pathologies
☐ Psychological pathologies
☐ Surgery
☐ Musculoskeletal disorder (e.g.: tendinitis, back pain, ...)
☐ None of the above

Do you think that your musculoskeletal disorder(s) may be due to your instrumental practice (or singing for singers)?

◯ Yes
◯ No

Lifestyle informations

To relax, you prefer:

☐ Playing sports
☐ Stretching and doing some relaxation
☐ Reading
☐ Doing creative activities
☐ Taking a walk
☐ Listening to music
☐ Watching movies / social media
☐ Doing activities with family / friends
☐ Taking a nap
☐ Cooking
☐ Other

Do you eat your meals regularly?

◯ Yes ◯ No

Do you feel that your diet is balanced?

◯ Yes ◯ No

What is your average amount of sleep per night during the week (excluding weekends)?

◯ 4 hours
◯ 5 hours
◯ 6 hours
◯ 7 hours
◯ 8 hours
◯ 9 hours
◯ 10 hours
◯ 11 hours
◯ 12 hours
◯ 13 hours
◯ 14 hours
◯ 15 hours
◯ 16 hours

Do you feel that the quality of your sleep is good?

◯ 1 - Yes ◯ 2 ◯ 3 ◯ 4 ◯ 5 - No

Do you have any addictive behaviors (e.g. tobacco, alcohol...)?

◯ Yes ◯ No ◯ Do not wish to answer

Habits related to musical practice

At what age did you start your main instrument (or singing for singers)?

◯ 2 years ◯ 3 years ◯ 4 years ◯ 5 years ◯ 6 years ◯ 7 years ◯ 8 years ◯ 9 years
◯ 10 years ◯ 11 years ◯ 12 years ◯ 13 years ◯ 14 years ◯ 15 years ◯ 16 years
◯ 17 years ◯ 18 years

Did you take a break from music?

◯ Yes ◯ No

Do you practice a second instrument?
(includes singing)

◯ Yes ◯ No

If yes, specify:

____

Do you practice a third instrument?
(includes singing)

◯ Yes ◯ No

If yes, specify:

____

In the two years preceding your arrival at the HEM, how many hours per day on average did you spend practicing your main instrument (or singing for singers)?

{ql16} hours

What is your academic level at the HEM?

◯ Bachelor 1
◯ Bachelor 2
◯ Bachelor 3
◯ Master 1
◯ Master 2

At present, how many hours do you practice your main instrument (or singing for singers), all activities combined, per day on average?

{ql18} hours

When you practice your main instrument (or singing for singers) how do you organize your breaks?

◯ I take a break every 30 minutes
◯ I take a break every hour
◯ I take a break every 2 hours
◯ I take a break every 3 hours or more
◯ I take irregular breaks

What are your preferred working hours for your main instrumental practice (or singing for singers)?

____
(e.g. : 14h - 17h / 20h - 22h)

What factors prevent you from working these hours?

{ql21} {ql21_libre}

When you practice music, do you do any warm-up and/or cool-down exercises?

◯ Yes, both
◯ Yes, warm-up exercises
◯ Yes, cool-down exercises
◯ No

Position used to practice your main instrument (or singing for singers), all activities combined:

{ql_assis} Sitting time {ql_debout} Standing time

Name, according to you, 3 causes of injuries or health problems related to the musician's musical practice. {ql23_1} {ql23_2} {ql23_3}

Do you see a health care professional regularly?

◯ Yes ◯ No

Is this health care professional a musicians' specialist?

◯ Yes ◯ No

What kind of health professional is it?

☐ Physician
☐ Physiotherapist
☐ Osteopath
☐ Psychologist
☐ Other

How many times do you see a physician on average per year?
{ql26bis_m} time(s)

How many times do you see a physiotherapist on average per year?
{ql26bis_phy} times

How many times do you see an osteopath on average per year?
{ql26bis_o} times

How many times do you see a psychologist on average per year?
{ql26bis_psy} times

Which one? {ql26bis_autre_lequel}

How many times a year on average?
{ql26bis_autre_combien} times

Have you been diagnosed by a health professional with a problem related to your musical practice?

◯ Yes, one ◯ Yes, two ◯ Yes, three or more ◯ No

If yes:

Which one: {ql28_lesquels}
When: month of {ql28_mois} year {ql28_annee}

If yes:

Which one: {ql28_lesquels_2}
When: month of {ql28_mois_2} year {ql28_annee_2}

If yes:

Which one: {ql28_lesquels_3}
When: month of {ql28_mois_3} year {ql28_annee_3}

How do you find out about musicians' health issues?

- [ ] Health professionals
- [ ] Social Media
- [ ] Internet
- [ ] Colleagues
- [ ] Associations
- [ ] Seminars, conferences
- [ ] With the teacher of my main instrument
- [ ] Instructors in physical practice at the HEM
- [ ] Other
- [ ] I don't get informed

Self-rated health

Page 3/9

SRH questionnaire

	Bad	Reasonable	Good
How would you rate your general health status?	○	○	○

	Worse	About the same	Better
How would you rate your general health status compared to 5 years ago?	○	○	○
How would you rate your health status compared to others in your age group?	○	○	○

	To a great extent	Partly	Not at all
Do you think your health prevents you from doing things you would like to do?	○	○	○

Physical activity

Page 4/9

We are interested in finding out about the kinds of physical activities that people do as part of their everyday lives. The questions will ask you about the time you spent being physically active in the last 7 days. Please answer each question even if you do not consider yourself to be an active person.
Please think about the activities you do at work, as part of your house and yard work, to get from place to place, and in your spare time for recreation, exercise or sport. Think about all the vigorous activities that you did in the last 7 days. Vigorous physical activities refer to activities that take hard physical effort and make you breathe much harder than normal. Think only about those physical activities that you did for at least 10 minutes at a time.

During the last 7 days, on how many days did you do vigorous physical activities like heavy lifting, digging, aerobics, or fast bicycling?

◯ No vigorous physical activities ◯ 1 ◯ 2 ◯ 3 ◯ 4 ◯ 5 ◯ 6 ◯ 7

How much time did you usually spend doing vigorous physical activities on one of those days?

{h_ai} hour(s) per day {min_ai} minute(s) per day
{nsp_ai}

Think about all the moderate activities that you did in the last 7 days. Moderate activities refer to activities that take moderate physical effort and make you breathe somewhat harder than normal. Think only about those physical activities that you did for at least 10 minutes at a time.

During the last 7 days, on how many days did you do moderate physical activities like carrying light loads, bicycling at a regular pace, or doubles tennis?
Do not include walking.

◯ No moderate physical activities ◯ 1 ◯ 2 ◯ 3 ◯ 4 ◯ 5 ◯ 6 ◯ 7

How much time did you usually spend doing moderate physical activities on one of those days?

{h_am} hour(s) per day {min_am} minute(s) per day
{nsp_am}

Think about the time you spent walking in the last 7 days. This includes at work and at home, walking to travel from place to place, and any other walking that you have done solely for recreation, sport, exercise, or leisure.

During the last 7 days, on how many days did you walk for at least 10 minutes at a time?

◯ No walking ◯ 1 ◯ 2 ◯ 3 ◯ 4 ◯ 5 ◯ 6 ◯ 7

How much time did you usually spend walking on one of those days?

{h_m} hour(s) per day {min_m} minute(s) per day
{nsp_m}

The last question is about the time you spent sitting on weekdays during the last 7 days. Include time spent at work, at home, while doing course work and during leisure time. This may include time spent sitting at a desk, visiting friends, reading, or sitting or lying down to watch television.

During the last 7 days, how much time did you spend sitting on a week day?

{h_as} hour(s) per day {min_as} minute(s) per day
{nsp_as}

Stress

Page 5/9

The following ten questions ask about how you have been feeling in the last four weeks. For each question, mark the circle under the option that best describes the amount of time you felt that way.

	None of the time	A little of the time	Some of the time	Most of the time	All of the time
In the last four weeks, about how often did you feel tired out for no good reason?	○	○	○	○	○
In the last four weeks, about how often did you feel nervous?	○	○	○	○	○
In the last four weeks, about how often did you feel so nervous that nothing could calm you down?	○	○	○	○	○
In the last four weeks, about how often did you feel hopeless?	○	○	○	○	○
In the last four weeks, about how often did you feel restless or fidgety?	○	○	○	○	○
In the last four weeks, about how often did you feel so restless you could not sit still?	○	○	○	○	○
In the last four weeks, about how often did you feel depressed?	○	○	○	○	○
In the last four weeks, about how often did you feel that everything was an effort?	○	○	○	○	○
In the last four weeks, about how often did you feel so sad that nothing could cheer you up?	○	○	○	○	○
In the last four weeks, about how often did you feel worthless?	○	○	○	○	○

The next few questions are about how these feelings may have affected you in the last four weeks.

In the last four weeks, how many days were you TOTALLY UNABLE to work, study or manage your day to day activities because of these feelings?

◯ 0 day
◯ 1 day
◯ 2 days
◯ 3 days
◯ 4 days
◯ 5 days
◯ 6 days
◯ 7 days
◯ 8 days
◯ 9 days
◯ 10 days
◯ 11 days
◯ 12 days
◯ 13 days
◯ 14 days
◯ 15 days
◯ 16 days
◯ 17 days
◯ 18 days
◯ 19 days
◯ 20 days
◯ 21 days
◯ 22 days
◯ 23 days
◯ 24 days
◯ 25 days
◯ 26 days
◯ 27 days
◯ 28 days

[Aside from those days], in the last 4 weeks, HOW MANY DAYS were you able to work or study or manage your day to day activities, but had to CUT DOWN on what you did because of these feelings?

◯ 0 day
◯ 1 day
◯ 2 days
◯ 3 days
◯ 4 days
◯ 5 days
◯ 6 days
◯ 7 days
◯ 8 days
◯ 9 days
◯ 10 days
◯ 11 days
◯ 12 days
◯ 13 days
◯ 14 days
◯ 15 days
◯ 16 days
◯ 17 days
◯ 18 days
◯ 19 days
◯ 20 days
◯ 21 days
◯ 22 days
◯ 23 days
◯ 24 days
◯ 25 days
◯ 26 days
◯ 27 days
◯ 28 days

In the last 4 weeks, how many times have you seen a doctor or any other health professional about these feelings? (Number of consultations)

◯ 0 consultation
◯ 1 consultation
◯ 2 consultations
◯ 3 consultations
◯ 4 consultations
◯ 5 consultations
◯ 6 consultations
◯ 7 consultations
◯ 8 consultations
◯ 9 consultations
◯ 10 consultations
◯ 11 consultations
◯ 12 consultations
◯ 13 consultations
◯ 14 consultations
◯ 15 consultations
◯ 16 consultations
◯ 17 consultations
◯ 18 consultations
◯ 19 consultations
◯ 20 consultations
◯ 21 consultations
◯ 22 consultations
◯ 23 consultations
◯ 24 consultations
◯ 25 consultations
◯ 26 consultations
◯ 27 consultations
◯ 28 consultations

In the last 4 weeks, how often have physical health problems been the main cause of these feelings?

◯ None of the time ◯ A little of the time ◯ Some of the time ◯ Most of the time ◯ All of the time

Music performance anxiety

Page 6/9

I generally feel in control of my life

◯ 6 - Strongly Disagree ◯ 5 ◯ 4 ◯ 3 ◯ 2 ◯ 1 ◯ 0 - Strongly Agree

I find it easy to trust others

◯ 6 - Strongly Disagree ◯ 5 ◯ 4 ◯ 3 ◯ 2 ◯ 1 ◯ 0 - Strongly Agree

Sometimes I feel depressed without knowing why

◯ 0 - Strongly Disagree ◯ 1 ◯ 2 ◯ 3 ◯ 4 ◯ 5 ◯ 6 - Strongly Agree

I often find it difficult to muster the energy to do things

◯ 0 - Strongly Disagree ◯ 1 ◯ 2 ◯ 3 ◯ 4 ◯ 5 ◯ 6 - Strongly Agree

Excessive worrying is a characteristic of my family

◯ 0 - Strongly Disagree ◯ 1 ◯ 2 ◯ 3 ◯ 4 ◯ 5 ◯ 6 - Strongly Agree

I often feel that life has not much to offer me

◯ 0 - Strongly Disagree ◯ 1 ◯ 2 ◯ 3 ◯ 4 ◯ 5 ◯ 6 - Strongly Agree

Even if I work hard in preparation for a performance, I am likely to make mistakes

◯ 0 - Strongly Disagree ◯ 1 ◯ 2 ◯ 3 ◯ 4 ◯ 5 ◯ 6 - Strongly Agree

I find it difficult to depend on other

◯ 0 - Strongly Disagree ◯ 1 ◯ 2 ◯ 3 ◯ 4 ◯ 5 ◯ 6 - Strongly Agree

My parents were mostly responsive to my needs

◯ 6 - Strongly Disagree ◯ 5 ◯ 4 ◯ 3 ◯ 2 ◯ 1 ◯ 0 - Strongly Agree

Prior to, or during a performance, I get feelings akin to panic

◯ 0 - Strongly Disagree ◯ 1 ◯ 2 ◯ 3 ◯ 4 ◯ 5 ◯ 6 - Strongly Agree

I never know before a concert whether I will perform well

◯ 0 - Strongly Disagree ◯ 1 ◯ 2 ◯ 3 ◯ 4 ◯ 5 ◯ 6 - Strongly Agree

Prior to, or during a performance, I experience a dry mouth

◯ 0 - Strongly Disagree ◯ 1 ◯ 2 ◯ 3 ◯ 4 ◯ 5 ◯ 6 - Strongly Agree

I often feel that I am not worth much as a person

◯ 0 - Strongly Disagree ◯ 1 ◯ 2 ◯ 3 ◯ 4 ◯ 5 ◯ 6 - Strongly Agree

During a performance, I find myself wondering if I'll make it to the end

◯ 0 - Strongly Disagree ◯ 1 ◯ 2 ◯ 3 ◯ 4 ◯ 5 ◯ 6 - Strongly Agree

Thinking about the evaluation I may get interferes with my performance

◯ 0 - Strongly Disagree ◯ 1 ◯ 2 ◯ 3 ◯ 4 ◯ 5 ◯ 6 - Strongly Agree

Prior to, or during a performance, I feel sick or faint or have a churning in my stomach

◯ 0 - Strongly Disagree ◯ 1 ◯ 2 ◯ 3 ◯ 4 ◯ 5 ◯ 6 - Strongly Agree

Even in the most stressful performance situations, I am confident that I will perform well

◯ 6 - Strongly Disagree ◯ 5 ◯ 4 ◯ 3 ◯ 2 ◯ 1 ◯ 0 - Strongly Agree

I am often concerned about a negative reaction from the audience

◯ 0 - Strongly Disagree ◯ 1 ◯ 2 ◯ 3 ◯ 4 ◯ 5 ◯ 6 - Strongly Agree

Sometimes I feel anxious for no particular reason

◯ 0 - Strongly Disagree ◯ 1 ◯ 2 ◯ 3 ◯ 4 ◯ 5 ◯ 6 -Strongly Agree

From early in my music studies, I remember being anxious about performing

◯ 0 - Strongly Disagree ◯ 1 ◯ 2 ◯ 3 ◯ 4 ◯ 5 ◯ 6 - Strongly Agree

I worry that a bad performance may ruin my personal development

◯ 0 - Strongly Disagree ◯ 1 ◯ 2 ◯ 3 ◯ 4 ◯ 5 ◯ 6 -Strongly Agree

Prior to, or during a performance, I experience increased heart rate like pounding in my chest

◯ 0 - Strongly Disagree ◯ 1 ◯ 2 ◯ 3 ◯ 4 ◯ 5 ◯ 6 - Strongly Agree

My parents almost always listened to me

◯ 6 - Strongly Disagree ◯ 5 ◯ 4 ◯ 3 ◯ 2 ◯ 1 ◯ 0 - Strongly Agree

I give up interesting performance opportunities due to anxiety

◯ 0 - Strongly Disagree ◯ 1 ◯ 2 ◯ 3 ◯ 4 ◯ 5 ◯ 6 - Strongly Agree

After the performance, I worry about whether I played well enough

◯ 0 - Strongly Disagree ◯ 1 ◯ 2 ◯ 3 ◯ 4 ◯ 5 ◯ 6 - Strongly Agree

My worry and nervousness about my performance interferes with my focus and concentration

◯ 0 - Strongly Disagree ◯ 1 ◯ 2 ◯ 3 ◯ 4 ◯ 5 ◯ 6 - Strongly Agree

As a child, I often felt sad

◯ 0 - Strongly Disagree ◯ 1 ◯ 2 ◯ 3 ◯ 4 ◯ 5 ◯ 6 - Strongly Agree

I often prepare for a concert with a sense of dread and impending disaster

◯ 0 - Strongly Disagree ◯ 1 ◯ 2 ◯ 3 ◯ 4 ◯ 5 ◯ 6 - Strongly Agree

One or both of my parents were overly anxious

◯ 0 - Strongly Disagree ◯ 1 ◯ 2 ◯ 3 ◯ 4 ◯ 5 ◯ 6 - Strongly Agree

Prior to, or during a performance, I have increased muscle tension

◯ 0 - Strongly Disagree ◯ 1 ◯ 2 ◯ 3 ◯ 4 ◯ 5 ◯ 6 - Strongly Agree

I often feel that I have nothing to look forward to

◯ 0 - Strongly Disagree ◯ 1 ◯ 2 ◯ 3 ◯ 4 ◯ 5 ◯ 6 - Strongly Agree

After the performance, I replay it in my mind over and over

◯ 0 - Strongly Disagree ◯ 1 ◯ 2 ◯ 3 ◯ 4 ◯ 5 ◯ 6 - Strongly Agree

My parents encouraged me to try new things

◯ 6 - Strongly Disagree ◯ 5 ◯ 4 ◯ 3 ◯ 2 ◯ 1 ◯ 0 - Strongly Agree

I worry so much before a performance, I cannot sleep

◯ 0 - Strongly Disagree ◯ 1 ◯ 2 ◯ 3 ◯ 4 ◯ 5 ◯ 6 - Strongly Agree

When performing without music, my memory is reliable

◯ 6 - Strongly Disagree ◯ 5 ◯ 4 ◯ 3 ◯ 2 ◯ 1 ◯ 0 - Strongly Agree

Prior to, or during a performance, I experience shaking or trembling or tremor

◯ 0 - Strongly Disagree ◯ 1 ◯ 2 ◯ 3 ◯ 4 ◯ 5 ◯ 6 - Strongly Agree

I am confident playing from memory

◯ 6 - Strongly Disagree ◯ 5 ◯ 4 ◯ 3 ◯ 2 ◯ 1 ◯ 0 - Strongly Agree

I am concerned about being scrutinized by other

◯ 0 - Strongly Disagree ◯ 1 ◯ 2 ◯ 3 ◯ 4 ◯ 5 ◯ 6 - Strongly Agree

I am concerned about my own judgement of how I will perform

◯ 0 - Strongly Disagree ◯ 1 ◯ 2 ◯ 3 ◯ 4 ◯ 5 ◯ 6 - Strongly Agree

I remain committed to performing even though it causes me great anxiety

◯ 0 - Strongly Disagree ◯ 1 ◯ 2 ◯ 3 ◯ 4 ◯ 5 ◯ 6 - Strongly Agree

Perfectionism

Page 7/9

Do you consider yourself a perfectionist?

◯ Yes
◯ No

People can exhibit varying degrees of perfectionism. In this questionnaire, we want to examine the reasons why people are perfectionists. To answer the questions presented below, refer to your field of study in music. For each question, begin the sentence with: "I am a perfectionist..."

	Does not describe me at all	Describes me very little	Describes me a little	Describes me moderately	Describes me quite a bit	Describes me a lot	Exactly describes me
...because it brings me closer to the first place and the concrete privileges that come with it	◯	◯	◯	◯	◯	◯	◯
...because I feel guilty when I cannot meet my success criteria	◯	◯	◯	◯	◯	◯	◯
...although I do not see what this gives me	◯	◯	◯	◯	◯	◯	◯
...because getting closer to perfection gives me a pleasant energy	◯	◯	◯	◯	◯	◯	◯
...because it allows me to avoid the disapproval of my relatives	◯	◯	◯	◯	◯	◯	◯
...because it allows me to avoid dangers or accidents that could harm me or my loved ones	◯	◯	◯	◯	◯	◯	◯
...because it allows me to be fully committed in what is important to me	◯	◯	◯	◯	◯	◯	◯
...because it allows me to be respected by others	◯	◯	◯	◯	◯	◯	◯
...because it gives me access to honors, first place, etc.	◯	◯	◯	◯	◯	◯	◯
...because it allows me to be appreciated by some people	◯	◯	◯	◯	◯	◯	◯
...even if I have no good reason to be	◯	◯	◯	◯	◯	◯	◯
...because it is a good way to realize my projects	◯	◯	◯	◯	◯	◯	◯

...because it reassures me not to break or damage my objects by accident	○	○	○	○	○	○	○
...because I am disappointed in myself when I do not act perfectly	○	○	○	○	○	○	○
...to show others what I am worth	○	○	○	○	○	○	○
...because at the end of the day, there is something to gain (e.g.: medal, award of excellence, prize, money, scholarship, etc.)	○	○	○	○	○	○	○
...because it allows me to feel emotions that I like	○	○	○	○	○	○	○
...because I make sure I do not cause problems that could harm me or my relatives	○	○	○	○	○	○	○
...to avoid disappointing some people	○	○	○	○	○	○	○
...because I feel pleasure when I surpass myself	○	○	○	○	○	○	○
...because it provokes pleasant sensations in me	○	○	○	○	○	○	○
...although it does not make any difference whether I engage in perfectionism or not	○	○	○	○	○	○	○
...because it allows me to reach my goals	○	○	○	○	○	○	○
...because I would blame myself for not doing things perfectly	○	○	○	○	○	○	○
...because if it's not perfect, I could lose my reputation	○	○	○	○	○	○	○

Fatigue

Page 8/9

We would like to know more about any problems you have had with feeling tired, weak or lacking in energy in the last month. Please answer ALL the questions by ticking the answer which applies to you most closely. If you have been feeling tired for a long while, then compare yourself to how you felt when you were last well. Please tick only one box per line.

	Less than usual	No more than usual	More than usual	Much more than usual
Do you have problems with tiredness?	○	○	○	○
Do you need to rest more?	○	○	○	○
Do you feel sleepy or drowsy?	○	○	○	○
Do you have problems starting things?	○	○	○	○
Do you lack energy?	○	○	○	○
Do you have less strength in your muscles?	○	○	○	○
Do you feel weak?	○	○	○	○
Do you have difficulties concentrating?	○	○	○	○
Do you make slips of the tongue when speaking?	○	○	○	○
Do you find it more difficult to find the right word?	○	○	○	○

In the last month

	Better than usual	No worse than usual	Worse than usual	Much worse than usual
How is your memory?	○	○	○	○

Personality traits

Page 9/9

I see myself as someone who ...	strongly agree	agree	neither agree nor disagree	disagree	strongly disagree	can't choose
is reserved	◯	◯	◯	◯	◯	◯
is generally trusting	◯	◯	◯	◯	◯	◯
does a thorough job	◯	◯	◯	◯	◯	◯
is relaxed, handles stress well	◯	◯	◯	◯	◯	◯
has an active imagination	◯	◯	◯	◯	◯	◯
is outgoing, sociable	◯	◯	◯	◯	◯	◯
tends to find fault with others	◯	◯	◯	◯	◯	◯
tends to be lazy	◯	◯	◯	◯	◯	◯
gets nervous easily	◯	◯	◯	◯	◯	◯
has few artistic interests (except in my music studies)	◯	◯	◯	◯	◯	◯

Exclusion de l'étude

Date de l'exclusion :

Raison :
